# Factors affecting calcium oxalate dihydrate fragmented calculi regrowth

**DOI:** 10.1186/1471-2490-6-16

**Published:** 2006-07-05

**Authors:** A Costa-Bauzá, J Perelló, B Isern, P Sanchis, F Grases

**Affiliations:** 1 Laboratory of Renal Lithiasis Research, University Institute of Health Sciences Research (IUNICS), University of Balearic Islands, 07122 Palma of Mallorca, Spain

## Abstract

**Background:**

The use of extracorporeal shock wave lithotripsy (ESWL) to treat calcium oxalate dihydrate (COD) renal calculi gives excellent fragmentation results. However, the retention of post-ESWL fragments within the kidney remains an important health problem. This study examined the effect of various urinary conditions and crystallization inhibitors on the regrowth of spontaneously-passed post-ESWL COD calculi fragments.

**Methods:**

Post-ESWL COD calculi fragments were incubated in chambers containing synthetic urine varying in pH and calcium concentration: pH = 5.5 normocalciuria (3.75 mM), pH = 5.5 hypercalciuria (6.25 mM), pH = 6.5 normocalciuria (3.75 mM) or pH = 6.5 hypercalciuria (6.25 mM). Fragment growth was evaluated by measuring increases in weight. Fragment growth was standardized by calculating the relative mass increase.

**Results:**

Calcium oxalate monohydrate (COM) crystals formed on COD renal calculi fragments under all conditions. Under pH = 5.5 normocalciuria conditions, only COM crystals formed (growth rate = 0.22 ± 0.04 μg/mg·h). Under pH = 5.5 hypercalciuria and under pH = 6.5 normocalciuria conditions, COM crystals and a small number of new COD crystals formed (growth rate = 0.32 ± 0.03 μg/mg·h and 0.35 ± 0.05 μg/mg·h, respectively). Under pH = 6.5 hypercalciuria conditions, large amounts of COD, COM, hydroxyapatite and brushite crystals formed (growth rate = 3.87 ± 0. 34 μg/mg·h). A study of three crystallization inhibitors demonstrated that phytate completely inhibited fragment growth (2.27 μM at pH = 5.5 and 4.55 μM at pH = 6.5, both under hypercalciuria conditions), while 69.0 μM pyrophosphate caused an 87% reduction in mass under pH = 6.5 hypercalciuria conditions. In contrast, 5.29 mM citrate did not inhibit fragment mass increase under pH = 6.5 hypercalciuria conditions.

**Conclusion:**

The growth rate of COD calculi fragments under pH = 6.5 hypercalciuria conditions was approximately ten times that observed under the other three conditions. This observation suggests COD calculi residual fragments in the kidneys together with hypercalciuria and high urinary pH values may be a risk factor for stone growth. The study also showed the effectiveness of specific crystallization inhibitors in slowing calculi fragment growth.

## Background

Calcium oxalate dihydrate renal calculi constitute the most prevalent and recurrent type of renal lithiasis [1,2]. They are usually associated with hypercalciuria, and on occasions with urinary pH values above 6.0 [3-7]. The use of extracorporeal shock wave lithotripsy (ESWL) to treat these renal calculi commonly gives excellent fragmentation results due to their fragility [8]. Nevertheless, the retention of post-ESWL fragments within the kidney is an important health problem, and a study of calcium stone patients found only 32% were stone-free 12 months after ESWL [9]. It appears that persistence and growth of fragments is common following ESWL [10-14]. *In vitro *[15-17] and *in vivo *[9] studies suggest that citrate [9,15,16] and phytate [17] can reduce residual post-ESWL calculi fragment growth or agglomeration. Despite those findings, however, there is a need for better understanding of the factors that contribute to stone growth following ESWL. Such knowledge will assist in designing methods for preventing such growth.

The present study belongs to a series examining the regrowth of residual post-ESWL calculi fragments in terms of calculi type, urinary conditions and presence of crystallization inhibitors. While a previous study examined regrowth of calcium oxalate monohydrate (COM) residual post-ESWL calculi fragments [17], the present study examined calcium oxalate dihydrate (COD) calculi fragments.

## Methods

The study used 48 spontaneously-passed post-ESWL fragments of COD calculi collected on the day of the ESWL procedure. Fragment selection proceeded according to the general protocol applied by our laboratory in the study of all renal stones. This methodology is based on a combination of optical stereomicroscopy, infrared spectrometry and scanning electron microscopy (SEM) equipped with an energy dispersive X-ray analyzer (EDS) [18]. All selected fragments had a very similar morphology which was representative of that observed in the majority of spontaneously-passed post-ESWL COD calculi fragments. Fragment sizes varied from 2 to 4 mm.

**Figure 1:**
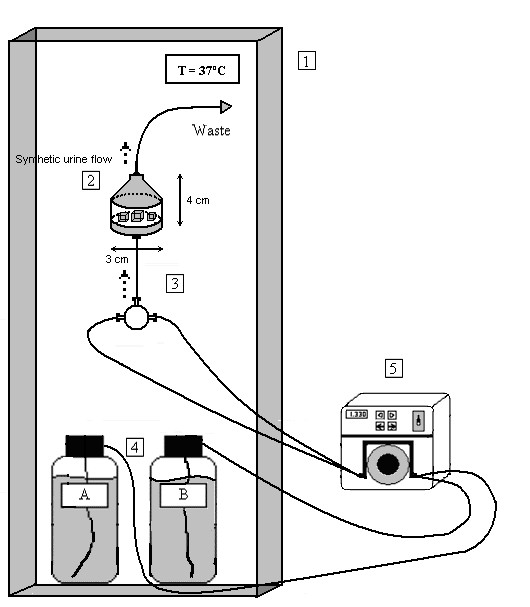
Diagram of the experimental flow device used for COD calculi crystallization studies. 1. Temperature-controlled chamber; 2. Flask containing post-ESWL calculi fragments; 3. Three-way T mixing chamber for A and B solutions; 4. A and B solutions for artificial urine; 5. Peristaltic pump.

Fragments were not pre-treated, and were placed into four hermetic flow chambers (3 cm diameter and 4 cm high), with each chamber containing 12 fragments. These chambers were then placed into a larger temperature-controlled (37°C) chamber. Each chamber was used to test a different incubation condition: pH = 5.5 and normocalciuria ([Ca total] = 3.75 mM), pH = 5.5 and hypercalciuria ([Ca total] = 6.25 mM), pH = 6.5 and normocalciuria ([Ca total] = 3.75 mM) and pH = 6.5 and hypercalciuria ([Ca total] = 6.25 mM). The duration of all incubations was 192 h, except for those under pH = 6.5 hypercalciuric conditions, which were for 48 h due to the high rate of fragment mass increase. The methodology used was similar to that previously described by Chow *et al*. [16,19]. Freshly prepared synthetic urine was introduced into the flow chambers using a multichannel peristaltic pump at a rate of 750 mL/day through the bottom of the flasks. The calculi fragments were placed on the porous flask bottom, allowing the entire fragment surface to be exposed to the artificial urine (see Figure 1). This system allows growth of new crystals on the fragments. Fragment growth was evaluated by measuring the difference in weight between the dried fragments before and after the experiment. The weight of the fragments was measured using a precision balance after the fragments had been kept in a desiccator until their weight became constant, indicating complete dryness. The mean growth rate of the 12 fragments under each condition was calculated. The growth of the different renal calculi fragments was standardized by calculating the relative mass increase in order to avoid the effects that different surface areas may have on growth rate measurements.

The effects of the crystallization inhibitors phytate, pyrophosphate and citrate were evaluated. The concentrations used corresponded to the physiological concentrations in urine.

The experimental research has been performed with the approval of the Bioethics Committee of the University of Balearic Islands and the research was carried out in compliance with the Helsinki Declaration.

### Synthetic urine

Synthetic urine supersaturated with calcium oxalate was prepared using a three-way T mixing chamber containing equal volumes of solutions A and B (compositions shown in Table 1). The pH of both solutions was adjusted either to 5.5 or 6.5. Solutions were stored for a maximum of 1 week at 4°C. Chemicals of reagent-grade purity were dissolved in deionized and redistilled water. All solutions were filtered through a 0.45 μm pore filter before use.

### Effect of crystal inhibitors

Compounds reported to inhibit crystal formation were added to the synthetic urine to the following final concentrations: 1.32–5.29 mM citrate as a sodium salt (supplied by Probus), 0.15–4.55 μM phytate as a sodium salt (supplied by Sigma), and 11.5–69.0 μM pyrophosphate as a sodium salt (supplied by Merck).

### Calcium-citrate complexation

Owing to the high concentration of citrate used, and considering its ability to complex with calcium ions, a calcium supplement was used in experiments involving citrate ions in order to achieve the same calcium oxalate supersaturation value as occurs in the absence of citrate. It must be noted that a decrease in supersaturation would imply a decrease in the crystallization rate that could not be assigned to inhibitory effects. The amount of calcium ions added was potentiometrically calculated using a calcium-selective electrode (Ingold) and a potentiometer (Crison 2002). Calcium standards in the presence and absence of citrate were prepared using synthetic urine as a matrix. The activity of free calcium ions must be the same in the presence and absence of citrate. Therefore, the calcium concentration was increased by 0.15 mM per 0.53 mM increase in the citrate concentration. The levels of phytate and pyrophosphate used were so low that the decrease in free calcium concentration was negligible, as determined potentiometrically. Consequently, it was not necessary to add a calcium supplement to the solutions containing phytate and pyrophosphate.

## Results

**Figure 2:**
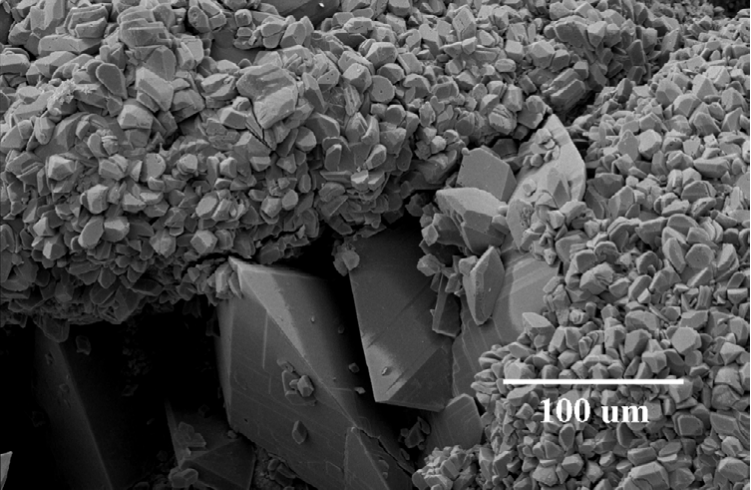
COM crystal formation on a post-ESWL COD renal calculus fragment following a 192 h incubation in normocalciuric (3.75 mM) and normooxaluric (0.28 mM) synthetic urine (pH = 5.5).

**Figure 3:**
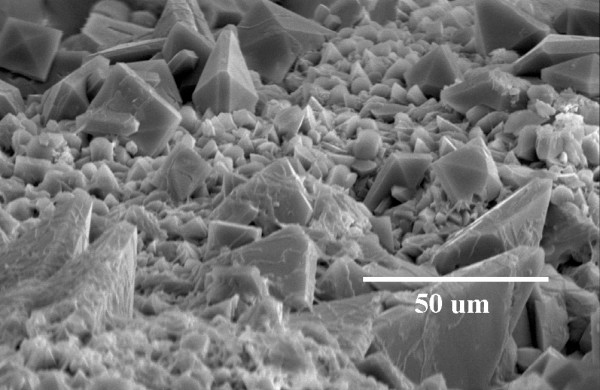
COM and COD crystal formation on post-ESWL COD renal calculi fragments following a 192 h incubation in hypercalciuric (6.25 mM) and normooxaluric (0.28 mM) synthetic urine (pH = 5.5).

**Figure 4:**
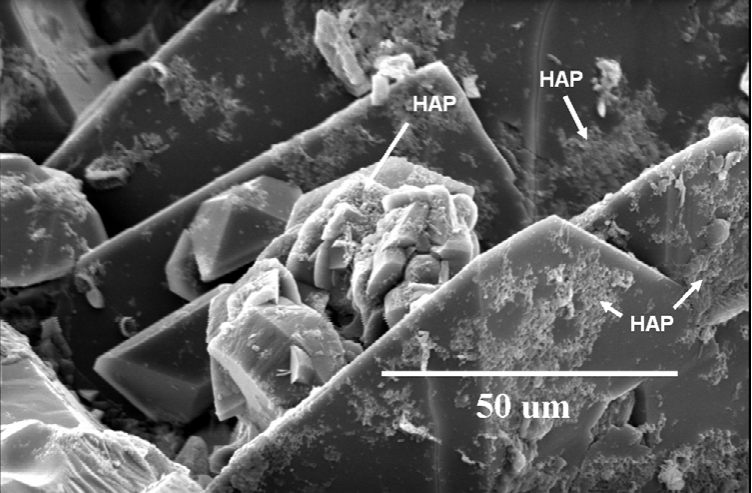
COM, COD and HAP crystal formation (arrows) on post-ESWL COD renal calculi fragments following a 192 h incubation in normocalciuric (3.75 mM) and normooxaluric (0.28 mM) synthetic urine (pH = 6.5).

**Figure 5:**
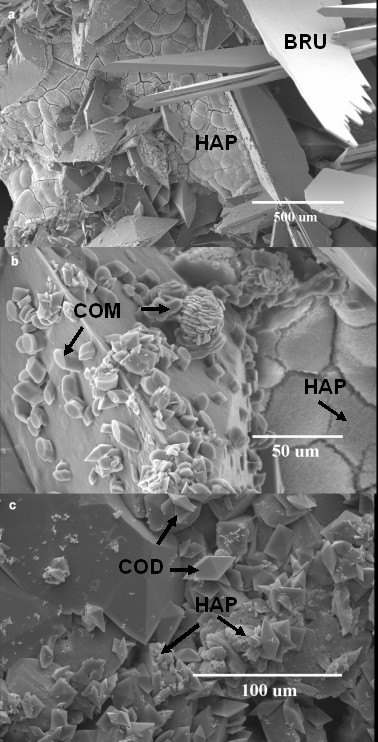
**a) **HAP and BRU crystal, **b) **COM and HAP crystal and **c) **COD and HAP crystal formation on post-ESWL COD renal calculi fragments following a 48 h incubation in hypercalciuric (6.25 mM) and normooxaluric (0.28 mM) synthetic urine (pH = 6.5).

Under all four incubation conditions, COM crystals were found to form on COD calculi fragments (Figures 2, 3, 4, 5). New COD crystal formation represented the minority (less than 50%) of crystal formation under pH = 5.5 hypercalciuric (Figure 3) and pH = 6.5 normocalciuric (Figure 4) conditions. In contrast, large amounts of COD crystals formed under pH = 6.5 hypercalciuric conditions (Fig 5a,c). Significant amounts of hydroxyapatite (HAP) and brushite (BRU) crystals formed under pH = 6.5 hypercalciuria conditions (Figure 5a,b), but not under other conditions.

The mean growth rates of COD calculi fragments under the four incubation conditions are summarized in Table 2. While growth rates of 0.22–0.35 μg/mg·h were observed under the majority of conditions, the rate was approximately ten times greater (3.87 ± 0.43 μg/mg·h) under pH = 6.5 hypercalciuria conditions.

**Figure 6:**
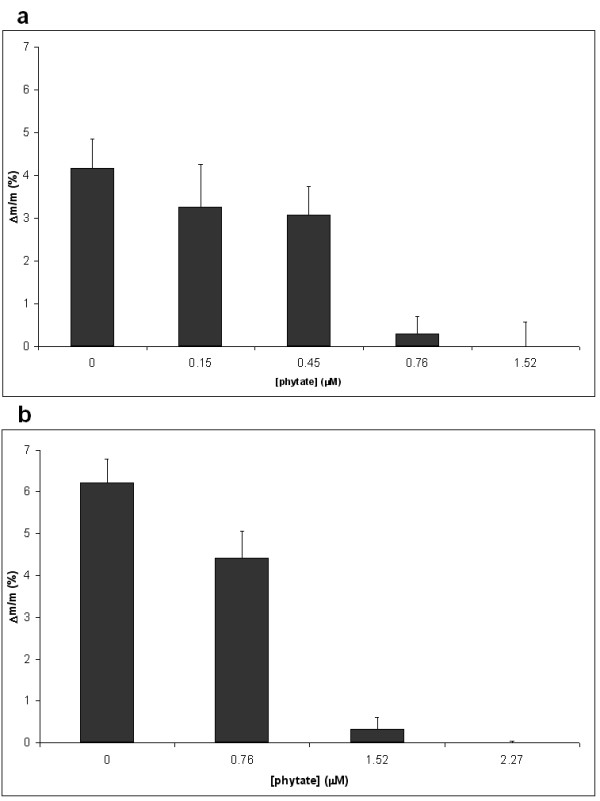
Increase in the relative weight of post-ESWL COD renal calculi fragments incubated for 196 h in normooxaluric (0.28 mM) synthetic urine at pH = 5.5 in the absence or presence of phytate. Values represent mean ± SEM for12 fragments. **a**. Normocalciuric urine ([Ca total] = 3.75 mM). **b**. Hypercalciuric urine ([Ca total] = 6.25 mM).

**Figure 7:**
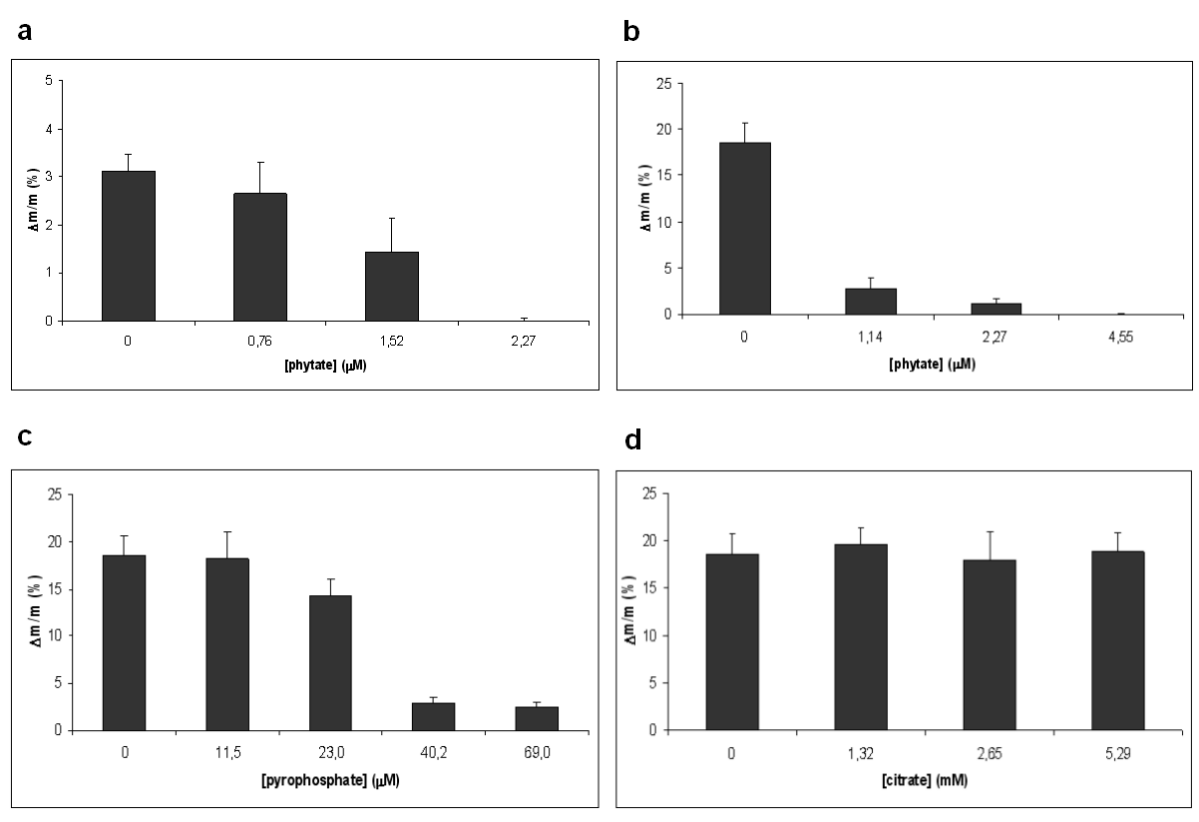
Increase in the relative weight of post-ESWL COD renal calculi fragments following incubation in normooxaluric (0.28 mM) synthetic urine at pH = 6.5. Values represent the mean ± SEM for 12 fragments. **a**. Incubation in normocalciuric urine ([Ca total] = 3.75 mM) for 192 hours in the absence or presence of phytate. **b**. Incubation in hypercalciuric urine ([Ca total] = 6.25 mM) for 48 hours in the absence or presence of phytate. **c**. Incubation in hypercalciuric urine ([Ca total] = 6.25 mM) for 48 hours in the absence or presence of pyrophosphate. **d**. Incubation in hypercalciuric urine ([Ca total] = 6.25 mM) for 48 hours in the absence or presence of citrate.

The effects of three known crystallization inhibitors (phytate, pyrophosphate and citrate) were investigated (Figures 6 and 7). We found that addition of 2.27 μM phytate to the pH = 5.5 hypercalciuria incubation, and the addition of 4.55 μM phytate to the pH = 6.5 hypercalciuria incubation completely inhibited the COD calculi fragment mass increase. Addition of lower phytate concentrations (less than 2.27 μM), mainly inhibited COM crystal formation. The addition of 69.0 μM pyrophosphate to the pH = 6.5 hypercalciuria incubation resulted in an 87% reduction of calculi fragment mass increase. The addition of 5.29 mM citrate to the pH = 6.5 hypercalciuria incubation had no effect on the COD calculi fragment mass increase.

## Discussion

The present study found that in normocalciuric/normooxaluric urine at pH = 5.5, only new COM crystals formed on COD calculi fragments. At the same pH, new COD crystals only formed under hypercalciuric conditions, in addition to COM crystals (Figure 3). Low phytate concentrations (less than 2.27 μM), inhibited this COM crystal formation. At pH = 6.5, COD crystals formed under normocalciuric conditions (Figure 4), while under hypercalciuria conditions the calcium phosphates HAP and BRU also formed with the COD crystals. These findings are consistent with several clinical observations showing that COM calculi are generally associated with a lack of crystallization inhibitors and COD calculi with hypercalciuria and high urinary pH values [3-6]. It is important to emphasize that the growth rate of COD calculi fragments was similar under all conditions except under pH = 6.5 hypercalciuria, conditions under which the growth rate was approximately ten times that observed under other conditions (see Table 2). These findings suggest that the presence of COD calculi fragments in the kidneys together with hypercalciuria and high urinary pH values (around 6.5) are a high risk factor for stone development. These data suggest that a COD calculus fragment of only 5 mg could become a 45 mg fragment in just 3 months under these conditions.

Crystallization inhibitors can notably retard calculi fragment development. The present study found that phytate at concentrations found in normal human urine [20] totally prevented COD calculi fragment growth. In addition, pyrophosphate also reduced the COD calculi fragment mass increase. In contrast, citrate did not show any significant crystallization inhibitory capacity under the conditions investigated. This finding appears to be in disagreement with previous reports showing that high citrate concentrations reduced the growth rate of stones by more than 50% [16]. However, this apparent discordance may be because the stated growth rate reduction must be assigned to citrate complexation with calcium, which results in an important reduction in the relative calcium oxalate supersaturation. Thus, it must be considered that in the present study a calcium supplement was added to obtain the same calcium oxalate supersaturation value found in the absence of citrate. Interestingly, while citrate did not inhibit calcium salt crystallization on COD crystals in the present study, it was shown to inhibit calcium salt crystallization on glycoproteins and organic matter [21,22]. These observations again highlight the specificity of crystallization inhibitors.

## Conclusion

The growth rate of COD calculi fragments under pH = 6.5 hypercalciuria conditions was approximately ten times that observed under other conditions. This observation suggests COD calculi residual fragments in the kidneys together with hypercalciuria and high urinary pH values may represent an important risk factor for stone development. In addition, the study found that specific crystallization inhibitors can effectively reduce calculi fragment growth.

## Competing interests

The author(s) declare that they have no competing interests.

## Authors' contributions

AC conceived the study and participated in the search and selection of COD calculi fragments. JP performed the crystallization studies under hypercalciuria conditions. BI performed the crystallization studies under normocalciuria conditions and pH = 5.5. PS performed the crystallization studies under normocalciuria conditions and pH = 6.5. FG participated in the study design and coordination, and assisted in preparation of the manuscript. All authors read and approved the final manuscript.

## Pre-publication history

The pre-publication history for this paper can be accessed here:



## Figures and Tables

**Table 1:** Composition of synthetic urine

**Solution A (mM)**		**Solution B (mM)**	
Na_2_SO_4_·10H_2_O	19.34	NaH_2_PO_4_·2H_2_O	15.45
MgSO_4_·7H_2_O	5.93	Na_2_HPO_4_·12H_2_O	15.64
NH_4_Cl	86.73	NaCl	223.08
KCl	162.60	Na_2_C_2_O_4_	0.57

Various volumes of a 1 M calcium solution (prepared by dissolving calcium carbonate with hydrochloric acid) were added to solution A to obtain final calcium concentrations ranging from 3.75–6.25 mM.

**Table 2:** Mean growth rates (relative mass increase, μg/mg·h) of post-ESWL COD renal calculi fragments under different incubation conditions. All incubations contained 0.28 mM oxalate. Results are expressed as mean ± SEM (n = 12).

**pH = 5.5**		**pH = 6.5**	
[Ca total] = 3.75 mM	[Ca total] = 6.25 mM	[Ca total] = 3.75 mM	[Ca total] = 6.25 mM
0.22 ± 0.04*	0.32 ± 0.03*	0.35 ± 0.05*	3.87 ± 0.43

* *p *< 0.05 vs. pH = 6.5 and [Ca total] = 6.25 mM conditions.

No significant differences were found between other groups
